# Engineered oncolytic virus coated with anti-PD-1 and alendronate for ameliorating intratumoral T cell hypofunction

**DOI:** 10.1186/s40164-025-00611-0

**Published:** 2025-02-15

**Authors:** Yufu Zhu, Xuefeng Zhang, Jiaqi Jin, Xiaoqian Wang, Yang Liu, Jian Gao, Diancheng Hang, Lin Fang, Hengzhu Zhang, Hongmei Liu

**Affiliations:** 1 Institute of Nervous System Diseases, Xuzhou Medical University, No.84 Huaihai West Road, Xuzhou, 221002 China; 2 Department of Neurosurgery, The Affiliated Hospital of Xuzhou Medical University, No.99 Huaihai West Road, Xuzhou, 221002 China; 3 Department of Biomedical Engineering, Southern University of Science and Technology, No. 1088, Xueyuan Avenue, Shenzhen, 518055 China; 4 Department of Neurosurgery, The Yangzhou Clinical Medical College of Xuzhou Medical University, Yangzhou University, No. 98, Nantong West Road, Yangzhou, 225009 China; 5 Department of Neurosurgery, The First Hospital of China Medical University, No. 155, Nanjing Bei Street, Shenyang, 110001 China; 6 Cancer Institute, Xuzhou Medical University, No. 209, Tongshan Road, Xuzhou, 221004 China

**Keywords:** Glioblastoma, Oncolytic viruses, Anti-PD-1, Alendronate, Tumor-associated macrophages

## Abstract

**Background:**

Glioblastoma is a highly aggressive and devastating primary brain tumor that is resistant to conventional therapies. Oncolytic viruses represent a promising therapeutic approach for glioblastoma by selectively lysing tumor cells and eliciting an anti-tumor immune response. However, the clinical efficacy of oncolytic viruses is often hindered by challenges such as short persistence, host antiviral immune responses, and T cell dysfunction.

**Methods:**

We have developed a novel therapeutic strategy by “dressing” oncolytic viruses with anti-PD-1 antibodies and alendronate (PD-1/Al@OV) to prevent premature clearance of the oncolytic viruses and enhance T cell function, thereby improving immunotherapy outcomes against glioma.

**Results:**

We found that in the high reactive oxygen species environment of the tumor, PD-1/Al@OV disassembled to release oncolytic viruses, anti-PD-1, and alendronate. The released anti-PD-1 blocked the PD-1/PD-L1 pathway, activating T cells; the alendronate eliminated tumor-associated macrophages, increasing the concentration of oncolytic viruses; and the oncolytic viruses directly lysed cancer cells, enhancing intratumoral T cell infiltration.

**Conclusion:**

This approach effectively improved the immunosuppressive microenvironment of glioblastoma and achieved a robust anti-tumor effect. Consequently, this study presents a novel strategy for immune combination therapy and the improvement of the glioblastoma immune microenvironment, thereby offering new prospects for the clinical application of oncolytic viruses.

**Supplementary Information:**

The online version contains supplementary material available at 10.1186/s40164-025-00611-0.

## Background

Glioblastoma (GBM) is a highly aggressive and devastating primary brain tumor, resistant to conventional therapies. Patients with GBM have a median survival time of approximately 15 months after undergoing multidisciplinary treatments, such as surgery and chemoradiotherapy [1, 2]. Therefore, there is an urgent need to develop novel and effective therapeutic strategies for GBM. OVs represent a new and promising treatment modality for GBM that has shown a certain curative effect [3]. OVs can selectively replicate in and kill cancer cells without causing severe effects on normal cells [4, 5]. Moreover, they can induce immunity and stimulate a robust anti-tumor immune response in GBM [6]. Clinical testing of OV therapy for GBM is currently underway, with promising safety results [7–10]. For instance, the oncolytic adenovirus Delta 24-RGD has completed a phase I study, demonstrating not only the safety and efficacy of this approach but also long-term survival in a subset of patients [11]. Other OVs, such as G207 and HSV1716, have been used in clinical research for patients with GBM in the USA and UK [7]. Furthermore, the triple-mutated oncolytic herpes virus G47∆ has been approved in Japan. This was done following a clinical trial [12]. However, the therapeutic efficacy of OVs alone is limited.

Recent studies have demonstrated that tumor-associated macrophages (TAMs) and microglia can limit the replication and spread of OVs [13, 14]. Macrophages can engulf and eliminate OVs, thereby reducing the viral titer in GBM. TAMs are important components of the GBM microenvironment and are considered a major source of relapse and chemoresistance. Approximately 30–50% of the cells in gliomas are TAMs, which facilitate the proliferation, survival, and migration of neoplastic cells [15]. Numerous studies reveal that TAMs promote glioma growth and invasion. In GBM, increased numbers of TAMs are associated with poor patient prognosis [16]. Reducing the presence of TAMs in gliomas results in attenuated glioma invasion and growth [17]. Our previous research also corroborates this finding [18]. Based on these studies, decreasing the presence of TAMs has been suggested as a potential therapeutic strategy to inhibit glioma growth and enhance the efficacy of OVs for GBM therapy. Alendronate, a bisphosphonate drug, can significantly inhibit tumor progression and angiogenesis by suppressing the proliferation and function of TAMs [19, 20].

OVs infection can induce a pro-inflammatory environment that stimulates T cell recruitment and destroys the physical barriers to T cell infiltration [21]. However, after OV treatment, the expression of the PD-L1 ligand at the immune checkpoint is reactively upregulated on tumor cells. This upregulation results in the dysfunction of recruited T cells and causes tumor resistance to oncolytic immunotherapy [22–24]. PD-1 is a receptor expressed on tumor-infiltrating lymphocytes within the cell membrane [25]. Its ligands are found on tumor-associated macrophages, dendritic cells, and tumor cells, as well as in the extracellular matrix. Once activated, these interactions can lead to T cell dysfunction or exhaustion, reducing cytokine production and thereby limiting their cytotoxic effects on tumor cells [26]. Blocking the interaction between PD-1 and its ligand restores T-cell function and promotes an anti-tumor immune response, making OVs ideal candidates for combination with immune checkpoint inhibitors [27]. Saha et al. reported that the effect of OV immunotherapy combined with an anti-PD-1 antibody in GBM was significantly better than that of OV immunotherapy alone [28]. Therefore, combining OVs with immune checkpoint inhibitors may be a promising therapeutic strategy for GBM patients.

**Fig. 1:**
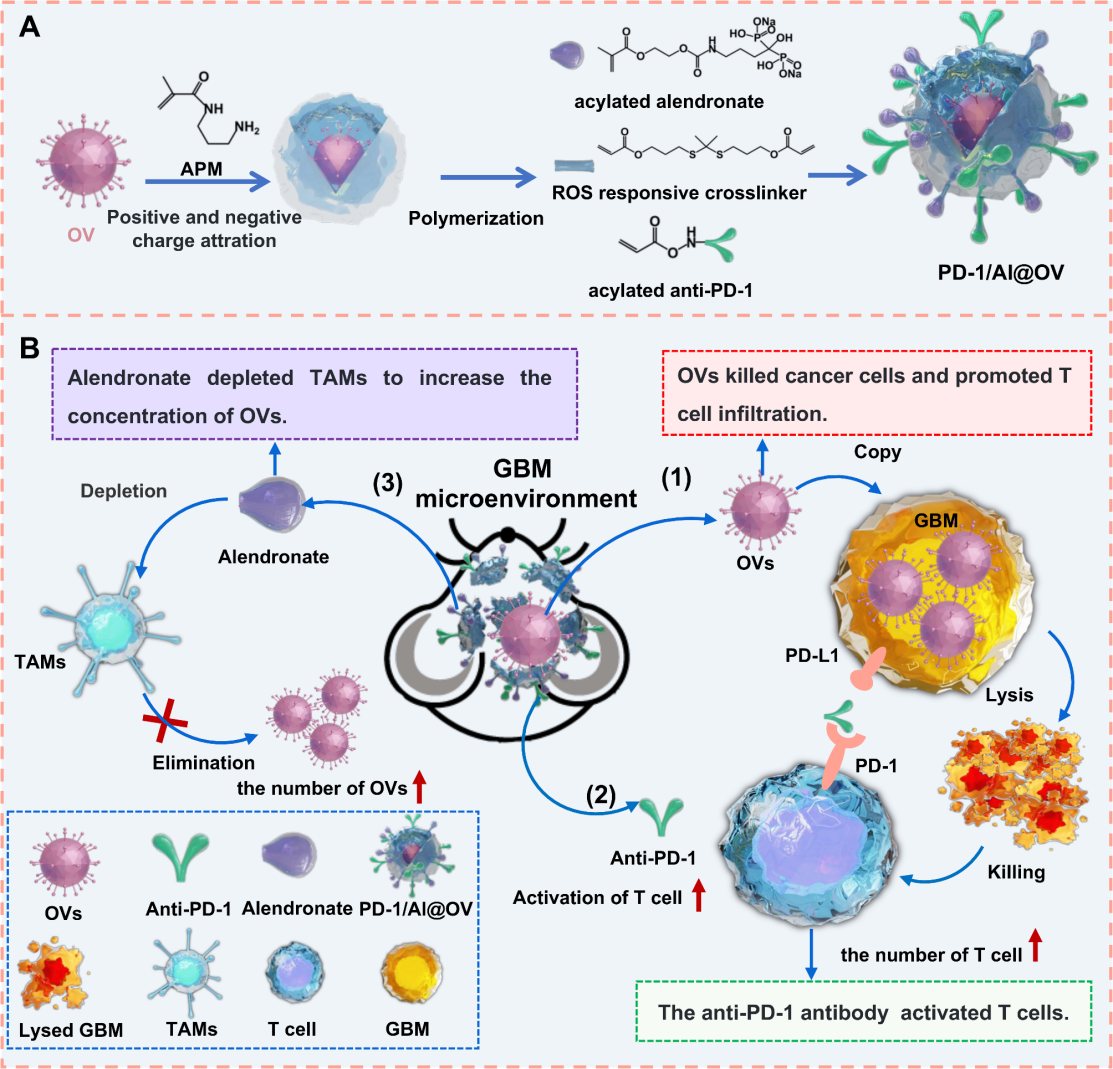
Schematic of the PD-1/Al@OV drug delivery system targeting GBM, TAMs, and T cells. (**A**) The formation process and mechanism of PD-1/Al@OV. (**B**) PD-1/Al@OV for GBM. (1) OVs directly lyse and kill GBM; (2) OVs can promote T cell infiltration; (3) Released anti-PD-1 can block the PD-1/PD-L1 pathway of T cells and enhance the therapeutic effect of T cells. (4) Released alendronate eliminates TAMs and increases the concentration of OVs

In this study, we developed a method to equip OVs with ROS-responsive anti-PD-1 and alendronate coatings (PD-1/Al@OV), thereby enhancing T cell activity and preventing OV engulfment by TAMs to improve therapeutic efficacy against GBM (Fig. 1). PD-1/Al@OVs were administered via in situ intracranial injection into orthotopic glioma xenograft mice. Subsequently, PD-1/Al@OVs disintegrated under high ROS tumor conditions to release anti-PD-1 and alendronate. PD-1/Al@OV offered four advantages: (1) OVs can directly lyse cancer cells to kill tumor cells; (2) OVs can promote intratumoral T cell infiltration; (3) Released anti-PD-1 can block the PD-1/PD-L1 pathway in T cells to enhance the therapeutic effect of OVs; (4) Released alendronate can eliminate TAMs, thereby increasing OV concentration. The feasibility of using PD-1/Al@OV against GBM was examined in detail through a series of in vitro and in vivo experiments.

## Methods

### Study design

The objective of this study was to develop a therapeutic strategy for GBM utilizing a nanovector of oncolytic adenovirus coated with alendronate and anti-PD-1, with the aim of depleting TAMs and enhancing the responsiveness and efficiency of immune checkpoint blockade (ICB) therapy. The in vivo antitumor effects were evaluated in GL261 and U87 tumor models. We followed up with mice across different treatment groups to validate the cytotoxic effects of PD-1/Al@OV on glioma cells, assessed the virus’s infectivity to glioma cells and its cytotoxicity, and conducted bioluminescence imaging to monitor tumor progression and to construct survival curves. The sample size was determined based on our previous experimental experience. Animals were randomly assigned to groups according to tumor size and body weight. Investigators were not blinded during the experimental procedures and outcome assessments. Euthanasia was carried out when animals exhibited signs of health deterioration. All experiments were conducted at least three times.

### Materials

The OVs used is a recombinant adenovirus with a deletion of the E1B-55kD gene, utilizing serotype 5 adenovirus as a backbone. The knob and shaft of serotype 35 adenovirus (Ad35) fiber protein replace those of serotype 5 adenovirus (Ad5), and EGFP or DsRed genes are inserted (Fig. S1). After amplification and purification, the titers expressing in plaque forming unit (PFU)/mL of these OVs were determined by half tissue culture infective dose (TCID50) method. 1,1′-Carbonyldiimidazole (Sigma-Aldrich 21860, USA), 2-Hydroxyethyl methacrylate (Sigma-Aldrich 128635, USA), Alendronate (TCI A2456, Japan), Dulbecco’s modified eagle medium (DMEM), (WISENT, 319-005-CL, Canada); Fetal bovine serum (WISENT, 086150, Canada); Annexin V-FITC/PI Apoptosis Detection Kit (BD Pharmingen, CB05850284, USA); cDNA Reverse Transcription Kit (Yeasen Biotechnology, 11119ES60, Shanghai China); CCK-8 Assay Kit (MedChemExpress, HY-K0301, USA); EdU Assay Kit (Ribo Biotechnology, C10310-1, China); IFN-γ ELISA Assay Kit (Absin, abs520007, China); IL-1β ELISA Assay Kit (Absin, abs520001, China); IL-6 ELISA Assay Kit (Absin, abs520004, China). The antibodies used in this study included: anti-CD46-APC (Biolegend, 352405, China), anti-CD45-BV650 (BD Pharmingen, 563410, USA), anti-F4/80-PE (BD Pharmingen, 565410, USA), anti-CD11b-FITC (BD Pharmingen, 554861, USA), FITC-conjugated anti-CD8a (BD Pharmingen, 553030, USA), anti-CD3 (Bio X cell, BE0002, USA), anti-PD-1 (Biolegend, 135233, USA), anti-CD8 (Bio X cell, BE0118, USA), anti-F4/80 (Bio X cell, BE0206, USA), anti-Foxp3 (Abcam, ab75763, UK), anti-CD46 (Absin, abs136051, China), anti-CAR (Thermo, PA5-12476, USA), anti-GAPDH (Proteintech, 60004-1-Ig, China), and anti-Ad5 (presented by associate professor Lin Fang, Xuzhou Medical University). TUNEL Kit (Meilunbio, MA0223, Dalian, China).

### Synthesis of acylated alendronate

Add carbonyldiimidazole (11 mmol, 1.1 equiv) and 150 mL freshly distilled acetonitrile to four-necked round-bottom flasks. Stir at room temperature for 1 h, and then add 2-hydroxyethyl methacrylate (10 mmol, 1 equiv) while continuing stirring. Leave the mixture at room temperature for 24 h. Add 50 mL of ethyl acetate and wash the organic layer with water and brine, dry with sodium sulfate, filter, and vacuum evaporate to separate the pure yellow oily compound. In a 10 mL round-bottom flask, dissolve allyl phosphorothioate (1 mmol, 1 equiv) previously adjusted to pH = 12 and freeze-dried, in 2 mL of water. Add imidazole carboxylate (1.1 mmol, 1.1 equiv) and adjust the pH to 12 using sodium hydroxide solution. Add the excess yellow oily compound synthesized earlier to the reaction. Wash the mixture three times with chloroform at pH = 4.5, concentrate the aqueous phase under vacuum, and precipitate the crude product in acetone to remove residual imidazole. Purify using reverse-phase C18 resin through column chromatography.

**Fig. 2:**
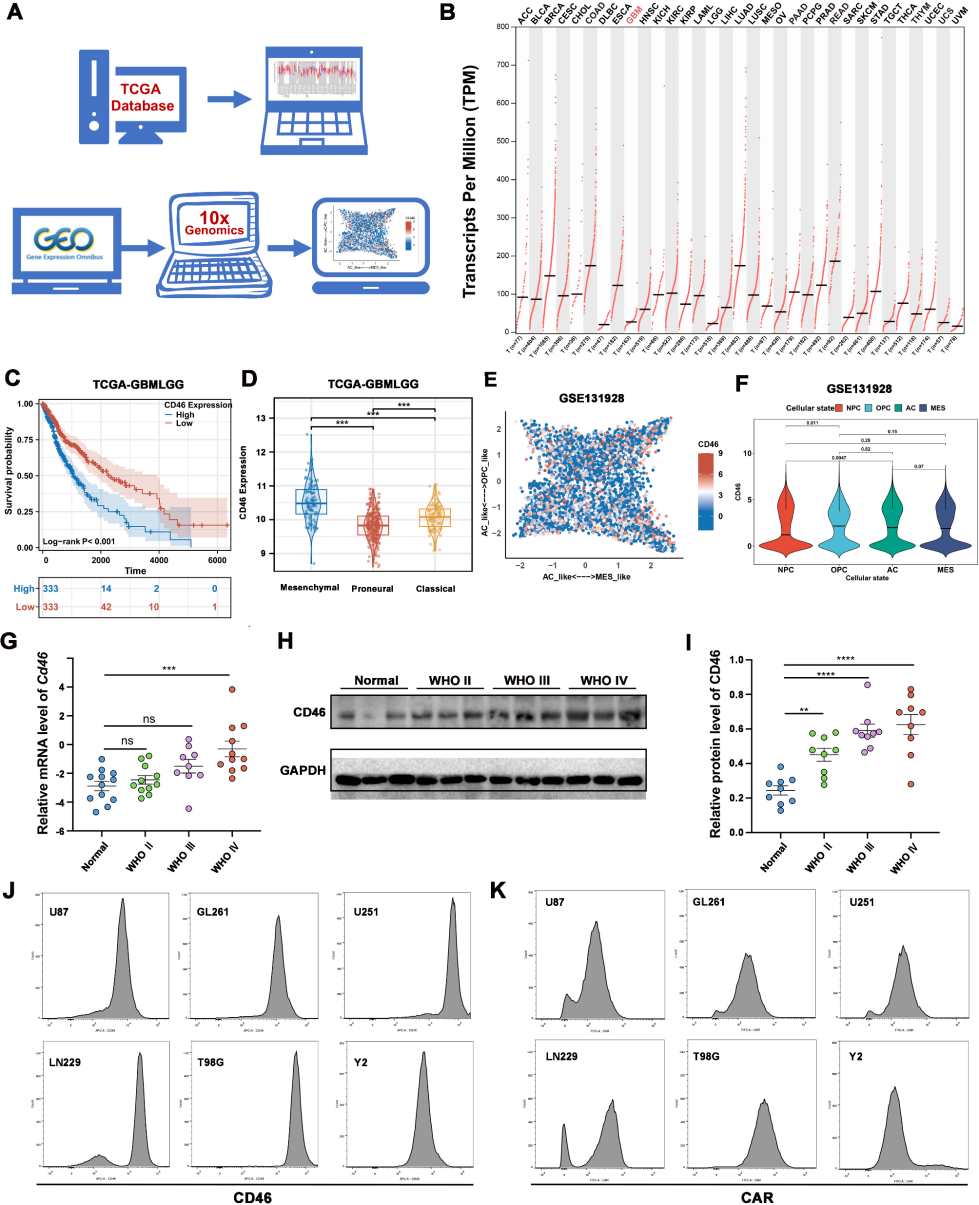
Physiochemical characteristics of the PD-1/Al@OV. (**A**) The formation process and mechanism of PD-1/Al@OV. (**B**) Size distribution of OVs. (**C**) Size distribution of PD-1/Al@OV. (**D**) TEM images of OVs under pH 7.4 PBS. (**E**) TEM images of PD-1/Al@OV under pH 7.4 PBS. (**F**) The zeta potential of OVs and PD-1/Al@OV. (**G**) The stability of the PD-1/Al@OV in CSF. In vitro alendronate (**H**) and anti-PD-1 (**I**) release profiles under pH 7.4 PBS or H_2_O_2_ conditions. Data were expressed as mean ± S.D., (*n* = 3)

The successful synthesis of acylated alendronate can be proved by ^1^H NMR detection of the synthesized product, and the yield is about 70%. ^1^H NMR (400 MHz, D_2_O) δ 6.09 (s, 1H), 5.68 (s, 1H), 4.48–4.17 (m, 4 H), 3.09 (t, *J* = 6.7 Hz, 2H), 1.94–1.79 (m, H) 5 H), 1.77–1.67 (m, 2H) (Fig. 2B); ^13^C NMR (100 MHz, D_2_O) δ169.5, 158.3, 135.6, 127.0, 73.9 (t, ^1^*J*_C−P_ = 127.3 Hz), 63.5, 62.7, 41.3, 31.3, 24.1 (t, ^2^*J*_C−P_ = 6.4 Hz), 17.3 (Fig. S2); ^31^P NMR (162 MHz, D_2_O) δ 18.53; HRMS (ESI) Calcd for C_11_H_20_NNa_2_O_11_P_2_ [M + H]^+^ 450.0301, found 450.0296 (Fig. S3).

### Synthesis of ROS responsive crosslinker

The Sm1 was dissolved in 40 mL of DMF, followed by the addition of K_2_CO_3_ and CH_3_I. The mixture was stirred at room temperature for 16 h, then 300 mL of water was added. The resulting mixture was extracted three times with ethyl acetate (EA) and the organic layer was subjected to a saturated sodium chloride back-extraction. After drying, the crude product was subjected to silica gel column purification using a gradient of petroleum ether (PE) and EA (PE: EA = 20:1 to 5:1) to yield a colorless oily liquid, referred to as Sm2. Sm2 was dissolved in 30 mL of dry tetrahydrofuran (THF) under a nitrogen atmosphere. To this solution, LiAlH_4_ was added dropwise at -20 °C and the mixture was stirred for 4 h. The reaction was quenched by adding 3 mL of water, followed by washing the solid three times with EA. After drying, the crude product was subjected to silica gel column purification using a gradient of PE and EA (PE: EA = 5:1 to 1:1) to yield a colorless oily liquid, referred to as Sm3. Sm3 was dissolved in 40 mL of dichloromethane (DCM) and then, at -20 °C, a solution of Sm2 and triethylamine (TEA) was added dropwise. The mixture was stirred at room temperature for 16 h, followed by washing with water three times. After drying, the crude product was subjected to silica gel column purification using a gradient of PE and EA (PE: EA = 30:1 to 10:1) to obtain the final ROS crosslinking agent. ^1^H NMR (500 MHz, DMSO-d6, δ, ppm) [δ6.41 (d, *J* = 12.2 Hz, 1H) (a), 6.10 (dd, *J* = 11.2, 10.3 Hz, 1H) (b), 5.83 (d, *J* = 10.3 Hz, 1H) (c), 4.23 (t, *J* = 6.3 Hz, 4 H) (d), 2.68 (t, *J* = 7.3 Hz, 4 H) (e), 1.96 ( p, *J* = 6.8 Hz, 3 H) (f), 1.59 (s, 4 H) (g)].

### Preparation and characterization of PD-1/Al@OV nanovectors

The acrylated N-succinimidyl ester (NSA) and anti-PD-1 were mixed at a molar ratio of 200:1 at room temperature, 800 rpm overnight to prepare the acylated anti-PD-1. OVs and N-(3-aminopropyl) methacrylamide (APM) 1 mM were reacted at 0 °C, 660 rpm, for 10 min. Then ROS responsive crosslinker, acylated anti-PD-1 (molar ratio of APM: complex 1, 12,000:1), and acylated alendronate (molar ratio of 500:1:1) were added and mixed for 5 min. The above solution was further reacted with persulfate (molar ratio of APS: complex 1, 500:1) and tetramethylethylenediamine (molar ratio of TEMED: APS, 2:1) under magnetic stirring at 0 °C for 2 h to initiate free radical polymerization.

### Detection of stability of PD-1/Al@OV in CSF

The prepared PD-1/Al@OV was placed into the cerebrospinal fluid of patients with hydrocephalus, and the hydrodynamic diameter was continuously measured for 7 days using a laser particle size analyzer (Zetasizer Nano ZS90, Malvern, UK) to assess the stability of PD-1/Al@OV in cerebrospinal fluid. This study was conducted with the fully informed consent of the patients and their families and was approved by the Ethics Committee of the Affiliated Hospital of Xuzhou Medical University (Ethics Number: XYFY2020-KL208-01).

### Drug release

To determine the amount of anti-PD-1 and alendronate released from PD-1/Al@OV, PD-1/Al@OV was dissolved in pH = 7.4 PBS or PBS containing 10 mM H_2_O_2_, and the released anti-PD-1 and alendronate were collected by dialysis catheter ultrafiltration at different time intervals. The released drugs were detected by using a multifunctional microplate reader. For anti-PD-1, we used the enzyme-linked immunosorbent assay (ELISA) method. The 96-well flat-bottom plate for ELISA included in the kit (Elabscience, Wuhan, China) was coated with 100 µL of 1 mg/mL recombinant PD-1 (GL Biochem, Shanghai, China) overnight at 4 °C. The next day, the plate was blocked with a blocking buffer. After incubation at 37 °C for 1 h, anti-PD-1 standards were prepared at different concentrations, and the samples to be tested were added to the wells. After incubation at room temperature for 1.5 h, the wells were washed 4 times, and 100 µL of peroxidase-labeled anti-mouse IgG was applied to the wells. The plate was then incubated at room temperature for 1 h. After incubation, the plate was washed 4 times, and the substrate solution was added. The optical density at 450 nm was measured using a multi-function microplate reader, and a standard curve was plotted based on the optical density values of the standards. The release of anti-PD-1 was calculated using the standard curve. The standard curve obtained was Y = 3.030 × X − 0.02639. For alendronate, we used the alendronate ELISA detection kit (Kejian Biology, China). A competitive ELISA method was employed, where the microplate was coated with alendronate-conjugated antigen. Alendronate standards or samples were added, and free alendronate competed with the pre-coated alendronate-conjugated antigen on the microplate strip for the alendronate antibody enzyme label. The TMB substrate was used for color development, and the color changed from blue to yellow after the addition of the stop solution. The absorbance was measured at 450 nm using a microplate reader, and the absorbance value was inversely proportional to the alendronate content in the samples. The release of alendronate was calculated using the standard curve. The standard curve obtained was Y = 3.167 × X + 0.004564.

### Cell culture

The human glioma cell lines U87, U87-GFP-Luci, U251, LN229 and T98G, the mouse glioma cell lines GL261 and GL261-GFP-Luci, the human astrocyte cell line HA1800, the mouse brain microvascular endothelial cell line bEnd.3, the mouse fibroblast cells line Y2 and the mouse monocyte/macrophage cell line Raw264.7 were all obtained from the Cell Bank of the Shanghai Branch of the Chinese Academy of Sciences. They were cultured in complete DMEM containing 10% fetal bovine serum and 1% penicillin-streptomycin. The cells were grown in a CO_2_ incubator at 37 °C.

### Database

TCGA data was obtained from the GlioVis data portal (http://recur.bioinfo.cnio.es/). The gene expression and clinical data in the GSE113510 dataset were acquired from the Gene Expression Omnibus (GEO) (https://www.ncbi.nlm.nih.gov/gds/). Single-cell RNA-seq data were extracted from GSE131928. The t-distributed stochastic neighbor embedding (tSNE) plot was executed using the Seurat package with R, version 3.6.0. The cell identities of each cluster were designated as previously explained. The level of CD46 was assessed by quantifying the Hallmark gene set (gene set M5923 taken from the Molecular Signatures Database (MSigDB)) utilizing the single-sample gene set enrichment analysis (ssGSEA) algorithm via the “GSVA” R package. The value obtained by ssGSEA indicated the relative abundance of CD46 in each sample. The median CD46 score was employed as the threshold value to categorize patients into high- and low-risk groups. The Kaplan-Meier survival curve and Cox regression analysis were created using the “survival” R package.

### Immunoblot assay

U87 or GL261 cells were seeded onto a 6-well plate. After the cells adhered to the plate, they were treated with PBS, OVs, and PD-1/Al@OV, respectively. For immunoblotting analysis, collected cells were lysed in RIPA lysis buffer, containing 50 mM Tris-HCl (pH 7.4), 150 mM NaCl, 5 mM EDTA, 10% glycerol, 1% NP-40, 0.1% SDS, 1 mM sodium fluoride, and 1 mM sodium orthovanadate. Cell proteins were boiled in loading buffer (Beyotime, P0015L, Shanghai, China). The primary antibodies used for immunoblotting were as follows: anti-Ad5 (provided by the Tumor Laboratory, Xuzhou Medical University), anti-CD46, and anti-GAPDH. For tissue samples, tissue homogenization was performed in RIPA lysis buffer, followed by the same steps as with cell. All immunoblot results were independently repeated for at least three times. Glioma specimens utilized in this study were derived from postoperative waste materials. The study has been approved by the Ethics Committee of the Affiliated Hospital of Xuzhou Medical University, with an exemption from patient informed consent, under the approval number XYFY2024-KL324-01.

### qRT-PCR

RNA was extracted from tumor tissues, and cDNA was synthesized using a cDNA reverse transcription kit according to the instructions. qRT-PCR reaction mixture was prepared (including 10 µL of SYBR Green Master Mix, 0.4 µL of PCR Forward, 0.4 µL of PCR Reverse, and 2 µL of cDNA template). The reaction was performed with 40 annealing cycles at 95°C for 10 seconds, 60°C for 34 seconds, preceded by denaturation at 95°C for 30 s. The relative expression levels of the target genes were quantitatively analyzed using GAPDH as the internal control. (CD46 qRT-PCR primers: Forward: 5’-ACCACTTTACACTCTGGAGC-3’, Reverse: 5’-GCTACCTGTCTCAGATGACG-3’; GAPDH qRT-PCR primers: Forward: 5’-ACCACAGTCCATGCCATCAC-3’, Reverse: 5’-TCCACCACCCTGTTGCTGTA-3’).

### Cell counting Kit-8 (CCK-8)

Logarithmic growth phase U87, GL261, HA1800, and bEnd.3 cells were prepared and counted at a density of 5 × 10³ cells per well, and then uniformly seeded in a 96-well plate. Control and experimental groups were set up, with 3–5 replicate wells per group. The cells were incubated in a CO₂ incubator until they adhered to the well. The old culture medium was aspirated, and different multiplicity of infection (MOI, PFU) at the indicated values of naked virus and drug-loaded virus were added to the corresponding wells of each group, followed by incubation for 48 h (MOI = the titer of OV × the corresponding volume/the number of cells). The old medium was removed, and 100 µL of a 10:1 diluted CCK-8 reagent was added to each well, followed by continued incubation until orange-yellow crystals appeared. The absorbance values were measured at a wavelength of 450 nm using an enzyme-linked immunoassay instrument. The experiment was repeated three times, and the data were statistically analyzed and the growth curve was plotted based on the absorbance of each cell culture well.

### 5-Ethynyl-2-deoxyuridine (EdU) cell proliferation kit

U87 and GL261 cells were pre-seeded in a 96-well plate at a density of 8 × 10³ cells per well, and then treated with naked virus or drug-loaded virus for 24 h in a CO₂ incubator. The old medium was aspirated, and a pre-prepared 50 µM EdU solution was added (100 µL per well), followed by incubation for 2 h in the incubator. The cells were fixed with 100 µL of 4% paraformaldehyde solution per well at room temperature for 30 min. Then, 100 µL of Apollo staining reaction solution was added to each well and incubated in the dark at room temperature on a decolorization shaker for 30 min. After cell staining with Apollo, 100 µL of Hoechst 33,342 reaction solution was added to each well and incubated in the dark at room temperature for 30 min. Images were captured and analyzed using a fluorescence inverted microscope after staining.

### ELISA assay

After preparing the orthotopic tumor model and treating it for 7 days, mice were intraperitoneally injected with 1% pentobarbital sodium and were deeply anesthetized. The mice were killed by decapitating, and the brain tissue was removed. The brain tumor area was precisely weighed and recorded. A tissue grinder was used for thorough grinding (in an ice-water bath), and after sufficient homogenization, the grinding solution was transferred to a centrifuge tube with a pipette. After centrifugation (4 °C, 12,000 rpm, 20 min), the supernatant was taken to detect the release of IL-1β, IFN-γ and IL-6 in the brain tumor area. The specific experimental steps were strictly carried out according to the instructions of the IFN-γ ELISA Assay Kit (Absin, abs520007, China), IL-1β ELISA Assay Kit (Absin, abs520001, China) and IL-6 ELISA Assay Kit (Absin, abs520004, China).

### Flow cytometry analysis

In a 6-well plate, U87 and GL261 cells were pre-seeded at a density of 5 × 10⁴ cells per well and then treated with naked virus or drug-loaded virus for 24 h. The cells were dissociated into single-cell status, washed twice, and centrifuged to remove PBS. The 10x Binding Buffer was diluted with PBS to prepare a 1x working solution. The cells from each sample were resuspended in 100 µL of the working solution, and 5 µL of Annexin V-FITC and 5 µL of Propidium Iodide were added to each tube. The cells were gently vortexed and incubated at room temperature in the dark for 15 min. Then, 400 µL of Binding Buffer was added to each tube for flow cytometry analysis.

### Animal

Male C57BL/6 mice were purchased from Jiangsu Gempharmatech Co., Ltd. (Nanjing, China). Male BALB/c nude mice were purchased from Zhejiang Weitonglihua Company (Zhejiang, China). All in vivo experiments were approved by the Ethics of Animal Committee of Xuzhou Medical University (approval number: 202402T002) and carried out under the guidance of Jiangsu Animal Protection Association. All experimental personnel have obtained the Jiangsu Provincial Laboratory Animal Professional Skills Certificate. All authors confirmed that the Ethics of Animal Committee of Xuzhou Medical University allowed the maximum tumor growth of mice to 10% of body weight after tumor implantationthe and that all mouse tumors did not exceed the maximal tumour burden.

### Orthotopic tumor model

Tumor transplantation animal model experiments were carried out according to our previous publication [29]. BALB/c nude mice were immobilized on a stereotactic apparatus and anesthetized with isoflurane inhalation to ensure complete anesthesia. Following confirmation, the heads of the mice were disinfected using iodine. A suitable incision was made along the midline of the posterior fontanelle using sterile scissors, and holes with a diameter of 3 mm were drilled at positions 1 mm posterior to the posterior fontanelle and 1.8 mm to the right. Under the guidance of the stereotactic apparatus, a microsyringe needle was inserted vertically into the brain striatum to inject 1 × 10^6^ U87 cells, establishing an orthotopic U87 glioma animal model. Similarly, 6 × 10^5^ GL261 cells were injected into the same location on the heads of C57BL/6 mice to establish an orthotopic GL261 glioma model. On the tenth day, the mice were randomly divided into the PBS, OV, OV + PD-1 + Al, Al@OV, PD-1@OV, and PD-1/Al@OV groups, and received a single treatment. Oral administration of alendronate was given. The OV + PD-1, Al@OV, PD-1@OV, and PD-1/Al@OV were intracranially injected into the mice. OV was administered intratumorally at a dose of 3 × 10^7^ PFU per mouse [30, 31]. Anti-PD-1 was administered intratumorally at a dose of 3 mg/kg per mouse [32]. For the number of mice in each group, 7 mice per group were used to measure the survival time of each group of mice after administration. The rest of the animal experiments had 3 mice per group. Tumor growth was monitored using an in vivo bioluminescence imaging system (IVIS; Berthold Technologies, LB 983 NC100, Germany). IVIS images were acquired 10 min after injecting luciferin (15 mg mL^− 1^, 10 mL kg^− 1^). Bioluminescent signals were analyzed using Living Image software (Caliper Life Sciences). Mouse survival was observed, and survival curves were plotted.

### Flow cytometry analysis of tumor-associated macrophages and T cell

On day 17 after in situ transplantation of GL261 cells, the mouse brain glioma region was dissected and homogenized in red blood cell lysis buffer. The resulting tissue lysate was then digested with 0.1% trypsin-EDTA to generate single-cell suspension. A certain quantity of cells was taken and resuspended in PBS (pH 7.4) by gentle shaking. BV650-conjugated anti-CD45 antibody was added to the cell suspension. Following that, FITC-labeled anti-CD11b and PE-labeled anti-F4/80 antibodies were used. After 15 min of staining in the dark at 4 ℃. Similarly, PerCP-Cy5.5-conjugated anti-CD3e, APC-Cy7-conjugated anti-CD4 and FITC-conjugated anti-CD8a were added. In the dark for 15 min at 4 °C, the T cells were detected on the machine. TAMs and T cells were detected on a flow cytometer. FlowJo v10.8.1 analysis software (FlowJo, USA) was utilized for data analysis.

### Immunohistochemistry

The slices were baked at 60 °C, dewaxed in xylene, rehydrated in graded ethanol, and then subjected to microwave antigen retrieval. The slices were treated with 3% hydrogen peroxide for 30 min, followed by overnight incubation at 4 °C with anti-CD3, anti-CD8, anti-F4/80, and anti-FOXP3 antibody. The bound antibody was detected with streptavidin peroxidase kit (Beijing Zhongshan Jinqiao Biological). Finally, counterstaining was performed. The number of glioma cells expressing positive staining in each field of view were quantified using the ImageJ software at a magnification of 40 times.

### Statistical analyses

All collected data were subjected to statistical analysis using GraphPad Prism 8.0 software. For pairwise comparisons of unpaired groups, an unpaired two-tailed Student’s t-test was conducted. For comparisons involving multiple groups, a one-way ANOVA test was employed. The groups being compared had similar variances. Survival estimates were calculated using the log-rank test of Kaplan-Meier analysis. The results were presented as bar graphs, displaying means ± standard deviation (S.D.), with statistical significance denoted as ns *P* > 0.05, *P < 0.05, **P < 0.01, ***P < 0.001, or ****P < 0.0001.

## Results

### CD46 is highly expressed in glioma and related to patient prognosis

**Fig. 3:**
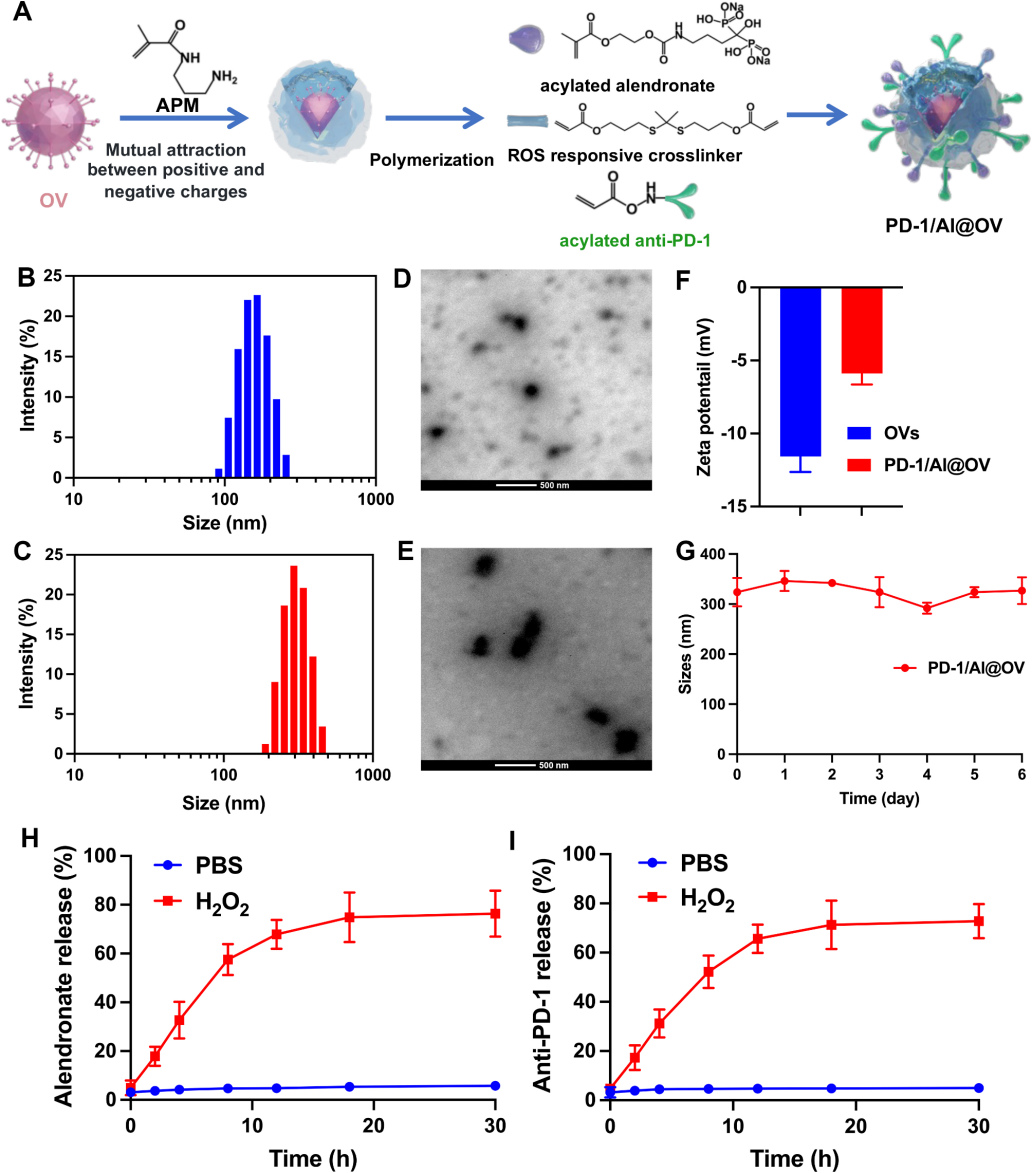
CD46 is highly expressed in glioma. (**A**) Schematic diagram of TCGA database and single-cell sequencing data analysis. (**B**) CD46 expression in tumor tissues for any gene of interest across all TCGA tumors. (**C**) The high CD46 expression group exhibited an unfavorable prognosis in patients of TCGA RNA-seq cohort (Low score: *n* = 333, High score: *n* = 333, log-rank test). (**D**) The CD46 expression is specifically higher in the mesenchymal group in TCGA RNA-seq cohort (Classical: *n* = 86, Mesenchymal: *n* = 96, Proneural: *n* = 238, one-way ANOVA test, ***P < 0.001, *P < 0.05). (**E**) Two-dimensional representation of cellular states. Each quadrant corresponds to one cellular state, the exact position of malignant cells (dots) reflect their relative scores for the meta-modules, and their colors reflect the density of cycling cells. (**F**) The analysis of Cell-state plots (Wilcoxon test). (**G**) qPCR was used to analyze the expression of CD46 mRNA in Normal, WHO II, III and IV grade glioma tissues, respectively, (Data are shown as mean ± S.D., Normal: *n* = 12, WHO II: *n* = 11, WHO III: *n* = 9, WHO IV: *n* = 11, one-way ANOVA test, ***P < 0.001, ns *P* > 0.05). (**H**) Immunoblotting of CD46 in Normal, WHO II, III and IV grade glioma tissues, respectively. (**I**) Quantitative analysis of CD46 protein expression in Normal, WHO II, III and IV grade glioma tissues, respectively. (Data are shown as mean ± S.D., *n* = 9, one-way ANOVA test, ***P < 0.001, **** *P* < 0.0001). (**J**) The expression of CD46 in different glioma cell lines was analyzed by flow cytometry. (**K**) Flow cytometry was used to analyze the expression of CAR in different glioma cell lines

The chimeric fiber Ad5/35 adenovirus was utilized, which facilitates infection via the specific recognition of the CD46 receptor on the surface of tumor cells, as glioma cells highly express CD46. To analyze the expression levels of CD46 in glioma patients, we conducted an analysis using the Cancer Genome Atlas Program (TCGA) database and single-cell data (GSE131928) as depicted in Fig. 3A. As shown in Fig. 3B, CD46 is highly expressed in tumor tissues of multiple cancer types, according to a pan-cancer analysis of the TCGA database, indicating that CD46 may serve as an oncogenic target across various cancer types. Kaplan-Meier survival analysis showed that high CD46 expression is associated with a poor prognosis in GBM patients (Fig. 3C). Furthermore, CD46 is highly expressed in mesenchymal GBM compared to proneural and classical GBM in the TCGA database (Fig. 3D) and single-cell sequencing analysis database (scRNA-seq, GSE131928) (Fig. 3E, F), suggesting that the expression of CD46 is associated with the maintenance of stem cells. GBM patients with a mesenchymal phenotype exhibit the poorest treatment response to chemotherapy. To investigate the expression profile of CD46 in glioma, we analyzed CD46 expression across different glioma grades. To assess the mRNA expression of CD46, quantitative real-time PCR (qRT-PCR) was employed. The mRNA expression level of CD46 was highest in WHO IV gliomas, which represent the most malignant form of the disease (Fig. 3G). To further examine the expression pattern of CD46 in glioma patients, we analyzed CD46 expression across different glioma grades. Total cell lysates were isolated from 27 human glioma tissue samples (9 WHO II, 9 WHO III, and 9 WHO IV, respectively), along with 9 non-tumor brain tissue samples. The expression level of CD46 protein in these tissues was subsequently assessed using WB. As illustrated in Fig. 3H and I, the expression of CD46 was significantly up-regulated in glioma tissues, particularly in high-grade glioma tissues (WHO III, WHO IV), when compared to normal brain tissue samples. Additionally, flow cytometry analysis (FCAS) was performed to examine the expression of CD46 (Fig. 3J) and the coxsackie-adenovirus receptor (CAR) (Fig. 3K) in different glioma cell lines. Compared with CAR expression, CD46 expression was high in U87, GL261, U251, LN299, T98G, and Y2. Thus, the expression of the primary receptor for Ad5 (CAR) is quantitatively deficient on glioma cells. These findings suggest that our chimeric fiber Ad5/35 adenovirus effectively targeted and eliminated glioma cells.

### Physiochemical characteristics of PD-1/Al@OV

To fabricate PD-1/Al@OV, a ROS-responsive crosslinker and acylated alendronate were synthesized. The synthetic paths of the acylated alendronate and ROS-responsive crosslinker are shown in Figs. S2 and S3. As shown in Figs. S4, S5, and S6, acylated alendronate was successfully synthesized, which was confirmed by ^1^H NMR, ^13^C NMR, and ^31^P NMR. The HRMS (ESI) calculated for C_11_H_20_NNa_2_O_11_P_2_ [M + H]^+^ was 450.0301, found 450.0296. The ROS-responsive crosslinker was also successful, as confirmed by ^1^H NMR spectroscopy (fig. S7). Subsequently, we prepared the ROS-responsive PD-1/Al@OV. OVs are negatively charged at physiological pH due to the abundant carboxyl residues from anionic peptides on their surface. Consequently, the positively charged monomer N-(3-aminopropyl) methacrylamide (APM) was absorbed onto the surface of OVs through electrostatic attraction and subsequently polymerized with acylated alendronate, ROS-responsive crosslinker, and acylated anti-PD-1 (Fig. 2A). The hydrated particle sizes and zeta potentials of PD-1/Al@OV nanovectors were measured using dynamic light scattering (DLS). After polymerization, the average size of PD-1/Al@OV increased from the initial 184.17 ± 12.89 nm (Fig. 2B) of naked OVs to 326.53 ± 6.30 nm (Fig. 2C). Furthermore, transmission electron microscope (TEM) images demonstrated that the particle size of PD-1/Al@OV (Fig. 2E) was larger than that of naked OVs (Fig. 2D), while maintaining the original monodisperse spherical structure of OVs. The surface zeta potential of PD-1/Al@OV increased to -5.9 ± 0.6 mV compared to the − 11.6 ± 0.9 mV of naked OVs (Fig. 2F). The stability of PD-1/Al@OV in cerebrospinal fluid (CSF) was evaluated. The findings indicated that PD-1/Al@OV remained stable in CSF, with no obvious alteration in the hydrodynamic diameter over a 7-day period (Fig. 2G). Glioma microenvironment-responsive release of anti-PD-1 and alendronate plays a crucial role, as anti-PD-1 activates CD8 + T cells and alendronate selectively depletes TAMs. Glioma is known for its high ROS environment [33]. To evaluate the ROS sensitivity of the prepared PD-1/Al@OV particles, we examined the time-dependent release of anti-PD-1 and alendronate from PD-1/Al@OV. As depicted in Fig. 2H and I, more than 76.4 ± 3.8% of alendronate and 72.8 ± 2.8% of anti-PD-1 were released within 12 h in the presence of H2O2, whereas the release was under 10% in PBS. These findings demonstrate that PD-1/Al@OV was successfully prepared and could release anti-PD-1, alendronate, and OVs in the glioma microenvironment.

### Infection capacity and anti-tumor effect of PD-1/Al@OV in vitro

**Fig. 4:**
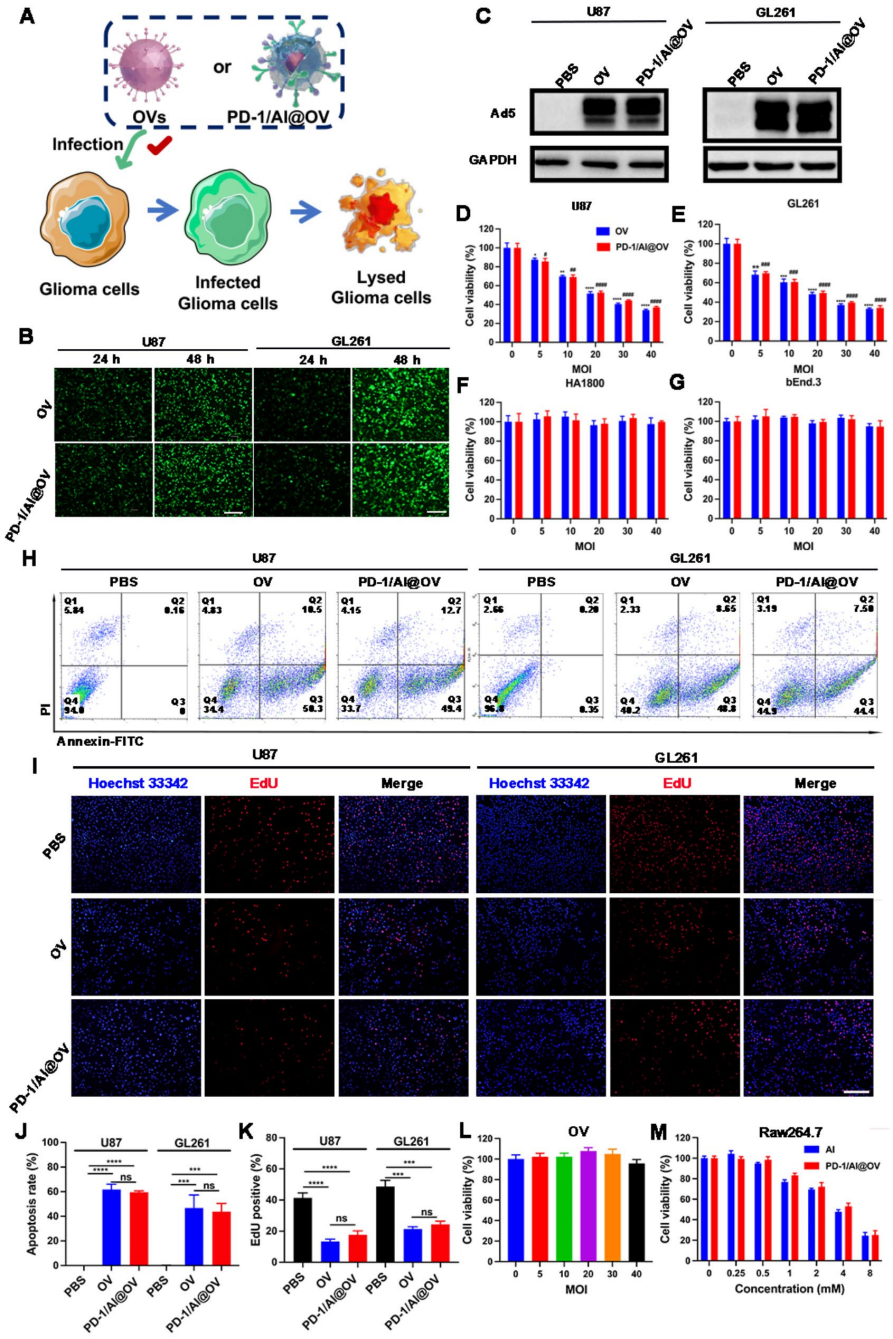
Infection capacity and anti-tumor effect of PD-1/Al@OV *in vitro.* (**A**) Schematic diagram of PD-1/Al@OV and OVs infection in glioma cells. (**B**) Fluorescence images of U87 and GL261 cells infected with PD-1/Al@OV. (**C**) Immunoblotting of Ad5 expression in U87 and GL261 cells after infection with OVs and PD-1/Al@OV. The cell viability assay of (**D**) U87 cells, (**E**) GL261 cells, (**F**) HA1800 cells, and (**G**) bEnd.3 cells after infection with OVs and PD-1/Al@OV at the indicated different MOI values. (**H**) FCAS analysis of apoptosis after OVs and PD-1/Al@OV treatment of U87 cells and GL261 cells. (**I**) EdU analysis of cell proliferation after OVs and PD-1/Al@OV infection of U87 cells and GL261cells. (**J**) Quantification of apoptosis in U87 cells and GL261 cells by FCAS. (**K**) Quantitative analysis of U87 cells and GL261 cells proliferation. (**L**) Raw264.7 cells after infection with OVs at the indicated different MOI values. Data are shown as means ± S.D., (*n* = 3). One-way ANOVA test. (**M**) Raw264.7 cells after infection with alendronate and PD-1/Al@OV at different titers. Data are shown as means ± S.D., (*n* = 3). *P < 0.05, ns*P* > 0.05, **P < 0.01, ***P < 0.001, ****P < 0.0001, one-way ANOVA test

To verify whether the anti-PD-1 and alendronate surface polymerization on OV (incorporating the EGFP gene) would affect the infectivity of OVs, we examined the infection of PD-1/Al@OV on U87, GL261, U251, and U373 glioma cells. After 24 and 48 h of incubation, PD-1/Al@OV exhibited similar infectivity to the OVs group (Fig. 4A, B, Fig. S8). The WB results further demonstrated that PD-1/Al@OV retained an infection capability similar to that of OVs (Fig. 4C). These results indicate that PD-1/Al@OV demonstrated a good infection ability, similar to naked OVs. The cytotoxicity of PD-1/Al@OV was evaluated using a CCK-8 assay. The indicated MOI (PFU) values of naked virus and drug-loaded virus were added to different cell lines. The data showed that both OVs and PD-1/Al@OV exerted dose-dependent cytotoxic effects on tumor cells (Fig. 4D and E), while exhibiting significantly lower cytotoxicity toward normal cells such as HA1800 (Fig. 4F) and bEnd.3 (Fig. 4G). This indicates that PD-1/Al@OV preferentially inhibits tumor cell growth. Furthermore, we evaluated the impact of PD-1/Al@OV on apoptosis and proliferation of glioma cells. FCAS using Annexin-V-FITC/propidium iodide double staining demonstrated that the percentage of apoptosis in U87 cells treated with PD-1/Al@OV (59.55 ± 1.17%) was nearly equivalent to that of OVs (61.82 ± 4.3%), whereas the control group had a significantly lower percentage of apoptosis (0.11 ± 0.04%) (Fig. 4H, J). Similarly, treatment with PD-1/Al@OV (43.78 ± 6.64%) or OVs (46.81 ± 10.54%) significantly increased the percentage of apoptosis in GL261 cells compared to the control group (0.41 ± 0.12%) (Fig. 4H, J). These outcomes indicate that PD-1/Al@OV efficiently induces apoptosis in glioma cells. Additionally, we evaluated the effect of PD-1/Al@OV on glioma cell proliferation using an EdU assay (Fig. 4I, Fig. S9A). Compared to the PBS group, both OVs and PD-1/Al@OV treatments significantly increased the number of EdU-positive cells, suggesting that PD-1/Al@OV could inhibit glioma proliferation in vitro (Fig. 4K, Fig. S9B). Alendronate can deplete TAMs [34]. In order to investigate whether PD-1/Al@OV had a depletion effect on TAMs, we used a CCK-8 test for detection. The results suggested that naked OVs had no effect on Raw264.7 activity (Fig. 4L). Both PD-1/Al@OV and the single drug alendronate inhibited Raw264.7 activity in a dose-dependent manner (Fig. 4M). These results indicated that PD-1/Al@OV could effectively eliminate TAMs and did not affect OVs’ ability to kill tumor cells.

### Detection of virus replication and antitumor effects of PD-1/Al@OV in C57BL/6J mice

**Fig. 5:**
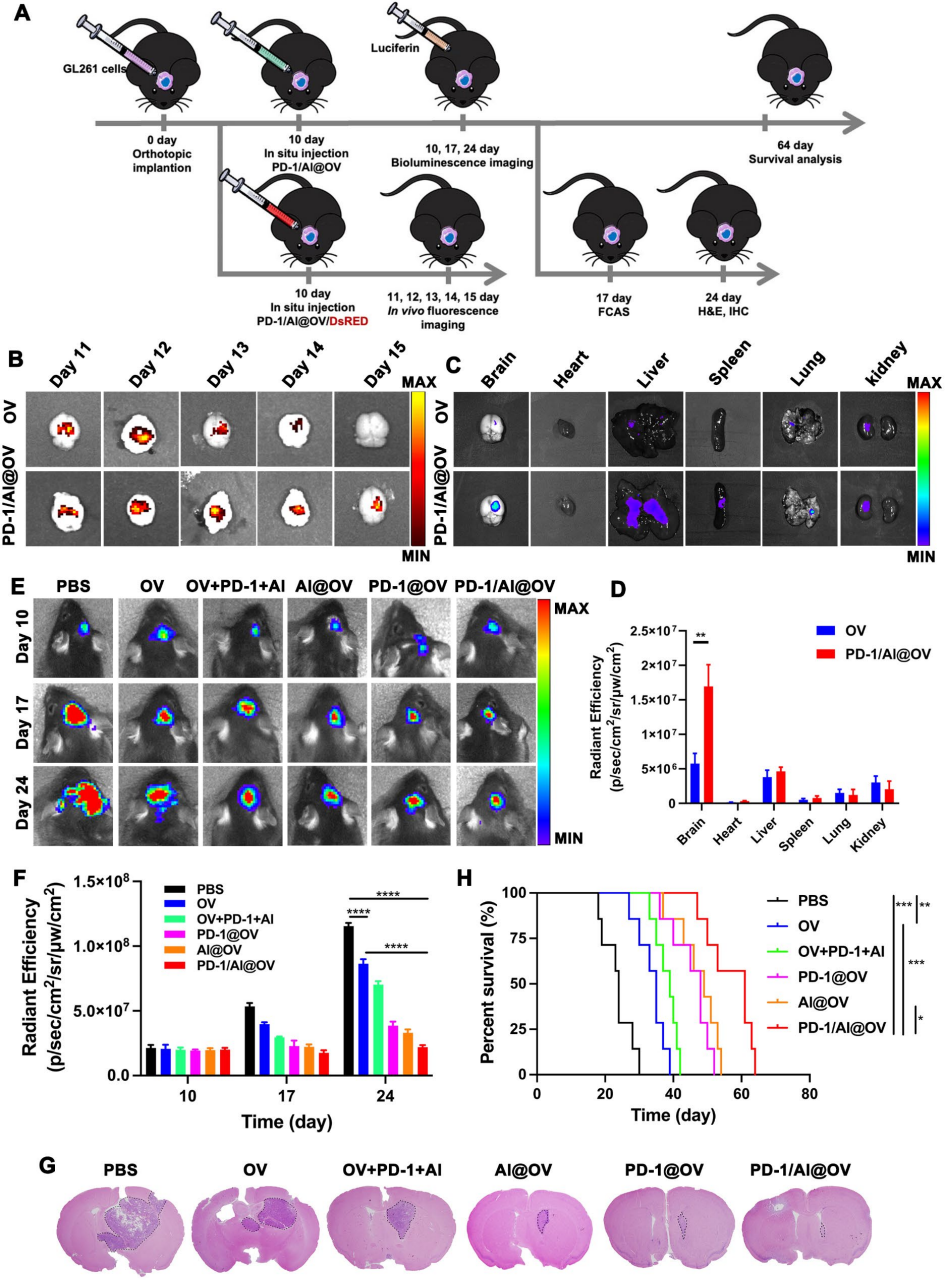
PD-1/Al@OV antitumor effects in C57BL/6J mice. (**A**) Experimental schedule of GL261-glioma inoculation and drug administration of PD-1/Al@OV. (**B**) IVIS images of the residence time of OVs and PD-1/Al@OV in glioma tissue. (**C**) Fluorescent image of OV/DsRed and PD-1/Al@OV/DsRed tissue distribution in vivo was observed with IVIS at post-injection 24 h. (**D**) Statistical analysis of fluorescence content of OVs and PD-1/Al@OV in mice tissues with imaging in vivo. Student’s t test. Data are means ± S.D., **P < 0.01. (*n* = 3). (**E**) Bioluminescence analysis of tumor size in mouse orthotopic xenograft model after 10, 17 and 24 days of treatment. (**F**) The bioluminescent quantification of xenografts derived. Data are shown as means ± S.D., (*n* = 3). ****P < 0.0001, one-way ANOVA test. (**G**) Representative H&E-stained images of coronal sections from mouse brains with orthotopic tumors. (**H**) Kaplan-Meier survival curves of mice implanted with GL261. *P < 0.05, **P < 0.01, ***P < 0.001, log-rank test. (*n* = 7)

To verify the residence time of OVs and PD-1/Al@OV in glioma tissues and their anti-tumor effects, we constructed an in situ GBM model using GL261 cells in C57BL/6J mice. The detailed therapeutic protocols for each group are outlined in Fig. 5A. We employed an in vivo animal imaging system (IVIS) to evaluate the replication and persistence of the virus, which was engineered with the DsRed red fluorescent gene. The experimental results indicated that PD-1/Al@OV effectively prolonged the duration of viral presence in tumor tissues compared to the naked virus (Fig. 5B) and distributed in the liver and kidneys (Fig. 5C, D and Fig. S10).

Based on the promising in vitro findings, the antitumor effect of PD-1/Al@OV in orthotopic models of GBM was assessed. The mice were randomly divided into PBS, OV, OV + PD-1 + Al, Al@OV, PD-1@OV, and PD-1/Al@OV groups and received intratumoral injections 10 days after tumor cell inoculation. At the indicated time points post-injection, the luciferase signal was monitored to assess tumor volume in each mouse (Fig. 5E). As expected, the PD-1/Al@OV group exhibited the highest antitumor efficacy, significantly reducing the luciferase signal (Fig. 5F). Brain tumor tissues were collected on day 24, and tumor-bearing brain slices were stained with hematoxylin and eosin (H&E) for imaging. Treatment with PD-1/Al@OV led to maximal tumor growth inhibition compared to all other treatments (Fig. 5G). TUNEL assay results demonstrated that PD-1/Al@OV significantly enhanced apoptosis in glioma cells (fig. S11). The median survival times for GL261-bearing mice were as follows: 35.0 days for the OV group, 39.0 days for the OV + PD-1 + Al group, 48.0 days for the PD-1@OV group, 49.0 days for the Al@OV group, and 61.0 days for the PD-1/Al@OV group (Fig. 5H). Mice treated with PD-1/Al@OV showed significantly longer survival times compared to all other treatment groups.

**Fig. 6:**
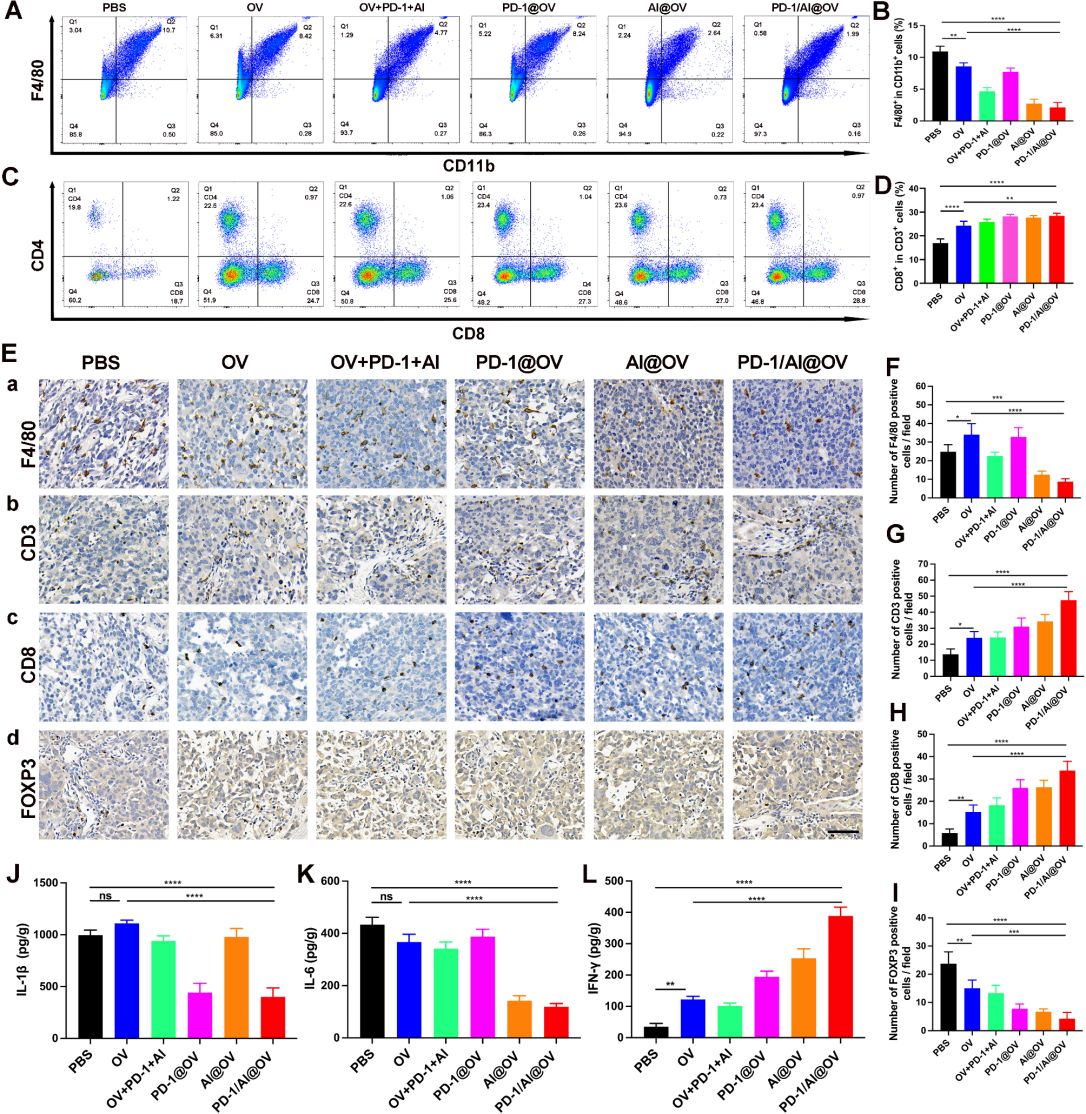
PD-1/Al@OV treatment enhance immune responses *in vivo.* (**A**) FCAS analysis of F4/80^+^ and CD11b^+^ cells in glioma tissue. (**B**) Quantitative analysis of TAMs cells in glioma by FCAS. (**C**) FCAS analysis of CD4^+^ and CD8^+^ T cells in glioma tissue. (**D**) Quantitative analysis of CD8^+^ T cells in glioma by FCAS. (**E**) (a-d) Immunohistochemical staining was used to observe the expression of F4/80, CD3, CD8 and FOXP3 in glioma tissues of mice orthotopic GL261 cells xenograft model 24 days after different drug treatment, Scale bar = 50 μm. (**F**) Quantitative analysis of F4/80 positive cells. (**G**) Quantitative analysis of CD3 positive cells. (**H**) Quantitative analysis of CD8 positive cells. (**I**) Quantitative analysis of FOXP3 positive cells. Levels of (**J**) IL-1β, (**K**) IL-6, (**L**) IFN-γ in tumor tissue as detected by ELISA 7 day after treatment. Data were expressed as mean ± S.D., (*n* = 3), one-way ANOVA test. *P < 0.05, **P < 0.01, **** *P* < 0.0001. **P < 0.01, ***P < 0.001, ****P < 0.0001, one-way ANOVA test. Data are shown as mean ± S.D., (*n* = 3)

Previous trials investigating OVs have uncovered that the efficacy of these viruses is partially due to their potential to awaken the immune system [35]. Therefore, we were motivated to investigate the impact of PD-1/Al@OV on the immune system in orthotopic GL261 gliomas in C57BL/6 mice. The effect of PD-1/Al@OV on TAM depletion and modulation of the glioma immune microenvironment was analyzed using flow cytometry (FCAS) and immunohistochemistry in GL261 glioma-bearing C57BL/6 mice. Treatment with PD-1/Al@OV significantly decreased the population of F4/80^+^CD11b^+^ macrophages compared to the PBS, OV, OV + PD-1 + Al, PD-1@OV, Al@OV, and PD-1/Al@OV groups (Fig. 6A, B). The infiltration of CD4^+^ and CD8^+^ cells was increased in the OV, OV + PD-1 + Al, PD-1@OV, Al@OV, and PD-1/Al@OV groups (Fig. 6C, D, Fig. S12). The flow cytometry gating strategy is depicted in Fig. S13. Similar results were observed in the immunohistochemistry analysis. The number of F4/80 + cells was decreased after PD-1/Al@OV treatment compared with that in their corresponding controls (Fig. 6Ea, F). The number of CD3^+^ cells and CD8^+^ cells was increased, and the number of Treg cells (FOXP3-positive cells) was significantly decreased after treatment with PD-1/Al@OV (Fig. 6Eb-d, G-I). To further demonstrate PD-1/Al@OV’s ability to modulate the immune microenvironment, we measured tumor-secreted IL-1β, IL-6, and IFN-γ levels using ELISA. IL-1β suppresses anti-tumor immune responses by promoting Tregs, IL-6 promotes tumor growth and metastasis while inhibiting anti-tumor immunity, and IFN-γ activates CD8^+^ cytotoxic T lymphocytes, enhancing their tumor-killing ability [36–38]. After PD-1/Al@OV treatment, IL-1β and IL-6 levels were significantly reduced, while IFN-γ expression increased in tumor tissues, indicating that PD-1/Al@OV effectively enhanced the anti-tumor immune responses (Fig. 6J-L). Therefore, we conclude that PD-1/Al@OV facilitates macrophage clearance and enhances T cell (CD3^+^ cells) infiltration in tumor tissue, particularly increasing cytotoxic T cell (CD8^+^ cells) infiltration while reducing Treg cells (FOXP3^+^ cells). These findings align with previous reports that alendronate depletes macrophages, OVs promote CD8^+^ T cell infiltration, and suppress tumor growth [39]. These results suggest that OVs loaded with anti-PD-1 and alendronate activate the anti-tumor immune response in vivo, and play a better role in inhibiting the growth of glioma and prolonging the survival time of tumor-bearing mice.

**Fig. 7:**
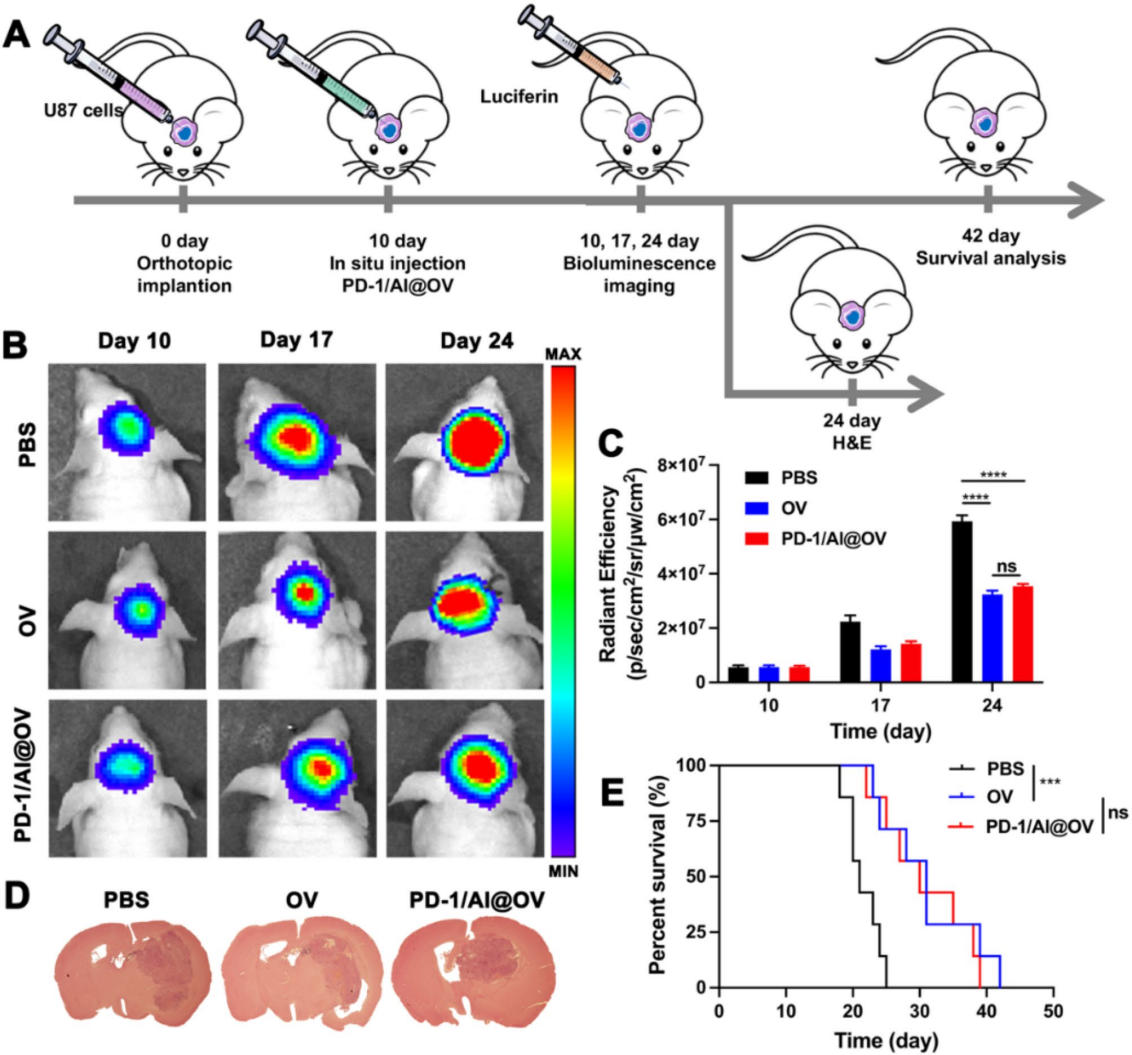
Detection of virus replication and PD-1/Al@OV antitumor effects *in vivo.* (**A**) Experimental schedule of U87-glioma inoculation and drug administration of PD-1/Al@OV. (**B**) Bioluminescence analysis of tumor size in mouse orthotopic xenograft model after 10, 17 and 24 days of treatment. (**C**) The bioluminescent quantification of xenografts derived. Data are shown as means ± S.D., (*n* = 3). ns*P* > 0.05, ****P < 0.0001, one-way ANOVA test. (**D**) Representative H&E-stained images of coronal sections from mouse brains with orthotopic tumors. (**E**) Kaplan–Meier survival curves of mice implanted with U87. ns*P* > 0.05, ***P < 0.001, log-rank test. Data are shown as means ± S.D., (*n* = 3) (*n* = 7)

To further validate whether PD-1/Al@OV leads to T cell infiltration and consequently enhances the efficacy of sensitizing immune checkpoint inhibitors, we injected U87/luci cells into BALB/c nude mice (which lack T cells) and measured tumor growth over time (Fig. 7A). As assessed via bioluminescence imaging on specific days, remarkable tumor suppression was observed in the PD-1/Al@OV treated group compared with the PBS group. However, the treatment with OVs or PD-1/Al@OV showed similar tumor suppressive effects (*P* > 0.05) (Fig. 7B, C). Brain tumor tissues were harvested on day 24, and tumor-bearing brain slices stained with H&E were imaged. The results confirmed a significant decrease in tumor burden in the PD-1/Al@OV group compared with the PBS group (Fig. 7D). Kaplan-Meier survival curves indicated that compared with the median survival time of the PBS group, which was 21 days, the median survival time for the OVs and PD-1/Al@OV groups was extended to about 30 days (Fig. 7E). From the above results, we found that there were no significant differences in antitumor effects between the OVs and PD-1/Al@OV groups, likely due to anti-PD-1 and alendronate having no therapeutic effects in immunodeficient BALB/c nude mice. As expected, in this model, drug-loaded viruses did not provide a significant benefit in inhibiting tumor growth and survival compared to naked virus therapy. These data underscore the importance of the immune response in the antitumor effect of drug-loaded viruses.

Finally, H&E staining was performed on slices of the main organs to evaluate toxicity in vivo. None of the groups showed any significant abnormalities. This indicates that PD-1/Al@OV has a reliable safety profile within the organism (Fig. S14). In addition, biochemical tests were carried out via tail vein blood collection, and there were no significant differences in the biochemical indices of ALT, AST, BUN, and CREA among any of the groups compared with the PBS group (*P* > 0.05) (Fig. S15A-D). The results again confirmed that PD-1/Al@OV had no significant toxic effects or side effects on normal tissues.

## Discussion

GBM is the most common and aggressive primary malignant brain tumor, accounting for 55% of all gliomas [40]. The current standard treatment involves surgical resection whenever possible, followed by adjuvant radiotherapy with concurrent temozolomide (TMZ) chemotherapy, and subsequent TMZ maintenance chemotherapy [41–44]. This conventional treatment approach not only yields poor outcomes but also damages normal tissue, lacking a tumor-selective therapeutic method that ultimately leads to patient suffering. To address the limitations of current treatment methods, a series of innovative treatment approaches have emerged. Among these, tumor immunotherapy stands out as it attempts to harness the innate cytotoxic abilities of the immune system to launch aggressive immune attacks against tumors and is regarded as a promising alternative therapy.

OVs are genetically attenuated or modified viruses that selectively infect and lyse tumor cells. This process results in the production of pathogen-associated chemokines and cytokines, the release of damage-associated molecular patterns (DAMPs), and the induction of tumor-associated antigens, which together trigger anti-tumor immune responses. As a result, OVs are considered a class of immunotherapeutic agents [45–47]. Gliomas, as the most representative “cold tumors” in immunotherapy, possess a unique immunosuppressive microenvironment that leads to inadequate efficacy of single OV therapy. Therefore, combination immunotherapy has garnered increasing attention [48–50]. Although OVs promote the infiltration of T lymphocytes into the tumor tissue during the process of oncolysis, the activated T cells express PD-1 receptors. These receptors can bind to the PD-L1 receptor on tumor cells, resulting in immune evasion by the tumor [51–53]. Furthermore, immune-suppressive cells such as TAMs play a dominant role in the tumor immune microenvironment. TAMs engulf and eliminate OVs, deplete T cell function by secreting immune-suppressive factors, and express PD-L1 and PD-L2 receptors [54, 55].

Based on the aforementioned circumstances, we prepared a ROS-responsive viral vector based on the negatively charged surface of OVs. This was achieved through a free-radical polymerization reaction. The vector was designed to deliver both anti-PD-1 and alendronate, enabling combined immune therapy. The goal was to recruit a greater number of T lymphocytes into tumor tissues and enhance their activity, thereby improving the immunosuppressive microenvironment of GBM and intensifying the antitumor immune response of the virus. This would help transition GBM immunotherapy from a “cold” to a “hot” state.

In our research project, we first examined the high expression of the CD46 target in GBM tissue samples, which was associated with poor prognosis in GBM patients. Subsequently, we synthesized PD-1/Al@OV using the free-radical polymerization reaction and evaluated its particle size, potential, and morphology. In an in vitro simulation of a high ROS environment, we confirmed its responsive release of anti-PD-1 and alendronate. At the cellular level, WB experiments demonstrated that PD-1/Al@OV could infect tumor cells. Further experiments including CCK-8, flow cytometry apoptosis, and EdU analysis proved that PD-1/Al@OV effectively inhibited GBM cells activity, promoted apoptosis, and did not affect normal cell growth. In vivo, PD-1/Al@OV effectively prolonged the duration of virus presence in tumor tissues compared to the naked OVs. In order to understand the GBM immune microenvironment, we constructed a GL261 cell immune-competent mouse model and performed immunohistochemical staining to evaluate immune cells within the tumor tissue. The results revealed that PD-1/Al@OV effectively eliminated TAMs, facilitated increased lymphocyte infiltration, and enhanced the antitumor immune response. Alendronate can achieve a certain therapeutic effect by inhibiting tumor-associated macrophages (TAMs) [19, 20]. Although TAMs are present in the U87 xenografts of nude mice, their immunosuppressive phenotype and lack of coordinated T-cell response limit the therapeutic effect of alendronate in this model. TAMs can secrete immunosuppressive factors and express ligands for inhibitory receptors such as PD-1, thereby suppressing T-cell effector functions [56, 57]. This immunosuppressive environment lacking T-cell immunity will significantly reduce the therapeutic effect of alendronate. In light of this, the lack of therapeutic superiority of PD-1/Al@OV over uncoated OV in the human U87 GBM xenografted nude Balb/c mouse model is consistent with the current understanding of the mechanism of action of alendronate.

Our research has already shown that the supplementary envelope of our investigational viral preparation did not affect the ability of the virus to infect cells. We will further explore the proliferation of the virus under encapsulated conditions in our subsequent research. By selecting cell lines suitable for viral growth for cell culture, we will observe the virus’s reproduction, measure viral titers, and monitor the expression of viral proteins. Additionally, by choosing appropriate animal models, we will observe the distribution and load of the virus in tissues. Through a multi-faceted approach to research methods, we will comprehensively assess the proliferation of the virus under encapsulated conditions. This will provide a deeper understanding for subsequent virological research and applications, optimize the structure and function of the virus under encapsulated conditions, and offer important research directions for the development of new anti-tumor therapeutic drugs and gene delivery systems.

## Conclusions

We successfully developed a method to let OVs wear ROS-responsive anti-PD-1 and alendronate clothes (PD-1/Al@OV) to enhance T cell activity and prevent the OVs from being engulfed by TAMs for improving immunotherapeutic efficacy against GBM. This combined immunotherapy approach effectively improved the immunosuppressive microenvironment of GBM and achieved a robust anti-tumor effect. Consequently, the findings of this study present a novel strategy for immune combination therapy and the improvement of the GBM immune microenvironment, thereby offering new prospects for the clinical application of OVs.

## Electronic supplementary material

Below is the link to the electronic supplementary material.

Supplementary Material 1: Figs. S1 to S15 for multiple supplementary figures.

## Data Availability

TCGA data was obtained from the GlioVis data portal (http://recur.bioinfo.cnio.es/). The gene expression and clinical data in the GSE113510 dataset were acquired from the Gene Expression Omnibus (GEO) (https://www.ncbi.nlm.nih.gov/gds/). Single-cell RNA-seq data were extracted from GSE131928.

## Abbreviations

Anti-PD-1: Anti-programmed cell death-1
OVs: Oncolytic viruses
Al: Alendronate
GBM: Glioblastoma
TAMs: Tumor-associated macrophages
ICB: Immune checkpoint blockade
Ad35: Serotype 35 adenovirus
Ad5: Serotype 5 adenovirus
DMEM: Dulbecco’s modified eagle medium
EA: Ethyl acetate
PE: Petroleum ether
THF: Tetrahydrofuran
DCM: Dichloromethane
TEA: Triethylamine
NSA: Acrylated N-succinimidyl ester
APM: N-(3-aminopropyl) methacrylamide
PBS: Phosphate buffered saline
CCK-8: Cell counting kit-8
EdU: 5-Ethynyl-2-deoxyuridine
WB: Western blot
CAR: Coxsackie-adenovirus receptor
DLS: Dynamic light scattering
TEM: Transmission electron microscope
CSF: Cerebrospinal fluid
H&E: Haematoxylin and eosin
FCAS: Flow cytometry analysis
TMZ: Temozolomide
